# MiR-9-3p augments apoptosis induced by H_2_O_2_ through down regulation of Herpud1 in glioma

**DOI:** 10.1371/journal.pone.0174839

**Published:** 2017-04-21

**Authors:** Ling Yang, Yongping Mu, Hongwei Cui, Yabing Liang, Xiulan Su

**Affiliations:** 1 Clinical Medicine Research Center, the Affiliated Hospital of Inner Mongolia Medical University, Hohhot, Inner Mongolia Autonomous Region, China; 2 Department of Clinical Laboratory, the Affiliated People’s Hospital of Inner Mongolia Medical University, Hohhot, Inner Mongolia Autonomous Region, China; Universitat des Saarlandes, GERMANY

## Abstract

MicroRNAs are short, single-stranded non-coding RNA molecules that function as regulators of tumor progression in various cancers, including glioma. The present study sought to investigate the biological functions of miR-9-3p in glioma progression. The results of a microRNA microarray indicated that microRNA-9-3p (miR-9-3p, miR-9*) is down-regulated in high-grade (grades III and IV) gliomas compared with non-tumor tissues. These results were confirmed with real-time PCR. The miR-9-3p expression level was associated with age and tumor grade. Herpud1 was regulated by miR-9-3p in glioma cells and tissues and was identified as a miR-9-3p target with luciferase reporter assays. Glioma cells transfected with miR-9-3p mimics or HERPUD1-RNAi had more apoptotic cells than them in control after induced by H_2_O_2_. Our results indicated that low expression of miR-9-3p results in a high level of Herpud1, which may protect against apoptosis in glioma.

## Introduction

Human gliomas are the most common malignant neoplasm of the central nervous system (CNS) in adults, accounting for 78% of intracranial primary tumors [1]. The mortality rate of glioma is also high [2]. Gliomas arise from neural cells and neural stromal cells (i.e., glial cells, ependymal cells, astrocytes and oligodendrocytes) [3]. The WHO (World Health Organization) classification system groups gliomas into four grades [4]. Although remarkable progress has been made in developing treatments to improve patient outcomes, including surgical resection, radiotherapy and chemotherapy, the current median survival time of patients with gliomas is no more than 11–15 months [5]. This poor prognosis is mostly due to limited knowledge of the molecular mechanisms of gliomas. Thus, it is essential to investigate the mechanisms involved in the progression of gliomas.

Reactive oxygen species (ROS) are generally defined as oxygen-containing small species, which are unstable and creating free radicals [6]. ROS can be either resulted from endogenous sources such as several mitochondrial mechanisms and inflammatory cascade or by exogenous sources such as radiation, chemicals and drugs, physiological changes such as aging [7–9]. High levels of ROS are harmful to cells, making damage to proteins, lipids and DNA, which results in mutation and cell death [10]. Excessive ROS have been found in most cancer cells [11], which suggested that cancer cells have developed an ability to survive oxidative stresses. ROS play a critical role in various signaling including hypoxia-inducible factor-1 alpha (HIF-1α), NFĸB, p53 and transforming growth factor beta (TGF-β)[6], which are involved in survival, proliferation, apoptosis, invasion, and metastasis[12,13]. Therefore, understanding the regulatory mechanisms of ROS in cancers might help in developing effective therapies against cancers, including gliomas.

Recent advances in epigenetics have revealed a class of small endogenous non-coding RNA-microRNAs (miRNAs) that are functionally important in human tumorigenesis [14]. Recently, many studies have demonstrated that deregulation of miRNAs can affect cell cycle control, apoptosis, invasion, migration, and resistance to chemotherapy and radiotherapy of gliomas [15–21]. Some miRNAs have been identified as oncomiRs [15,16], whereas others have been identified as tumor-suppressor miRNAs [17–19]. Studies have showed that miRNAs also involved in ROS regulation in many types of cancers. ROS decrease expression of let-7 family [22] and miR-34a [23]. While, ROS increase the expression of miR-21[24], miR-146a[25], miR-200s family[26], of which, miR-21 and miR-200s family in turn stimulates ROS production[24,26]

In this study, miRNA expression profiles from snap frozen samples of glioma were examined. We first identified a distinct pattern of miRNA expression in primary glioma tissues compared with normal brain tissues. Of the miRNAs with notable fold changes (fold change >1.5), three (miR-9-3p, miR-126, and miR-572) were selected for validation with real-time PCR. We ultimately identified Herpud1 as the target for miR-9-3p. MiR-9-3p enhanced H_2_O_2_ induced apoptosis through down regulation of Herpud1 in glioma cells. Our results suggested that miR-9-3p and its target Herpud1 may play an important role in the pathogenesis of glioma.

## Materials and methods

### Human tissue samples

Three fresh glioma tissues and 3 normal tissues (as controls) were used for genome-wide microarray. These samples were kindly providing from Beijing Tian Tan Hospital by Prof. Song Lin. Normal tissues were from healthy individuals who had experienced traffic accidents, trauma, or hematencephalon. These tissues were snap frozen in liquid nitrogen after resection and stored at -80°C until use for genome-wide microarray screening. Before the study, glioma tissue samples were re-classified according to WHO criteria [4]. Glioma diagnoses were independently confirmed by two professional pathologists and were summarized in Table 1.

**Table 1:** Clinical characteristics of microarray study subjects.

No.*	Age (year)	Gender (male/female)	Type	Stage
1	37	male	Glioblastoma	IV
2	46	female	Anaplastic oligodendroglioma	III
3	52	male	Anaplastic astrocytoma	III
4	28	male	Normal	N/A
5	35	male	Normal	N/A
6	41	female	Normal	N/A

* 1–3 patients, 4–6 control; N/A: not applicable

A total of 36 paraffin-embedded glioma tissues and 5 normal tissues (sap fresh frozen) were collected from the Department of Pathology or Neurosurgery of the Affiliated Hospital of Inner Mongolia Medical University. Among them, twenty four (grade III, IV) paraffin-embedded glioma tissues and 5 normal tissues (for calibration) were used to validate the microarray. Twenty four glioma tissues of grade III, IV and 12 glioma tissues of grade I, II were used for analyzing the association between the expression levels of the miR-9-3p and clinicopathological features. The normal brain tissue samples used as controls were obtained from patients who had experienced traffic accidents, trauma, or hematencephalon. Tissues were snap frozen in liquid nitrogen after resection and stored at -80°C until use. All of the tissues were collected by the Department of Pathology or Neurosurgery of the Affiliated Hospital of Inner Mongolia Medical University from February 2009 to March 2012. The present study was approved by the Investigation and Ethics Committee of the Affiliated Hospital of Inner Mongolia Medical University. All participants gave written informed consent, and all specimens were handled anonymously according to the ethical and legal standards of the Helsinki Declaration.

### MicroRNA expression profiling

A total of 6 samples were randomly chosen for an initial genome-wide microarray screening. An mParaflo miRNA microarray assay was performed by an external service provider (LC Sciences, Houston, TX). A starting amount of 5 mg total RNA was size-fractionated using a YM-100 Microcon centrifugal filter (GE Millipore, Billerica, MA), and the 3’ ends of the small RNAs (300 nt) were extended with poly(A) tails by using poly(A) polymerase. An oligonucleotide tag was ligated to each poly(A) tail for fluorescent dye staining. RNA samples were labeled with two different tags for the dual-sample experiments.

Hybridization was performed overnight on an mParaflo microfluidic chip using a microcirculation pump. Each detection probe on the microfluidic chip consisted of a chemically modified nucleotide coding segment complementary to the target miRNA (1145 human miRNA [hsa-miRNA] entries in version 8.1 from miRBase, http://microrna.sanger.ac.uk/sequences/) or other control RNAs. Detection probes also contained a spacer segment of polyethylene glycol to extend the coding segment away from the substrate. The detection probes were generated by in situ synthesis using photogenerated reagent chemistry. The hybridization melting temperatures were balanced by chemical modifications of the detection probes. Hybridization was performed in 100 mL of 66SSPE buffer (0.90 M NaCl, 60 mM Na_2_HPO_4_, 6 mM EDTA, pH 6.8) containing 25% formamide at 34°C. After hybridization, the miRNAs were detected through fluorescence labeling using tag-specific Cy3 and Cy5 dyes. Hybridization images were collected using a laser scanner (GenePix 4000B, Molecular Device) and digitized using Array-Pro image analysis software (Media Cybernetics). Data were analyzed by first subtracting the background and then normalizing the signals using a locally weighted regression filter. For two-color experiments, the ratio of the two sets of detection signals (log2 transformed, balanced) was calculated, and the significance of the differences was determined by t-test. Significantly different signals were those with p values less than 0.05. Multi-array normalization and clustering analysis were performed using a hierarchical method, with an average linkage and Euclidean distance metric. Clustering plots were generated for those miRNAs with a total signal density of more than 1000 using MultiExperiment Viewer software (v4.0, 2006) from The Institute for Genomic Research.

### Cell culture and nucleofection

Human glioma cell line U251 and 293t were cultured in DMEM (HyClone, Logan, UT, USA) supplemented with 10% fetal bovine serum (FBS, HyClone). All cells were incubated at 37°C under 5% CO_2_. Negative control miRNAs, miR-9-3p mimics were purchased from Ribobio (Guangzhou, China). Three HERPUD1-RNAi sequences “GTGATACAAATTGGTGAACAACTCGAG TTGTTCACCAATTTGTATCAC”, “CAAGTGATGGTTTAAGGCAAACTCGAG TTTGCCTTAAACCATCACTTG” and “CACGACAGTACTACATGCAATCTCGAG ATTGCATGTAGTACTGTCGTG” were constructed into GV102 plasmid, respectively. The control sequence was “TTCTCCGAACGTGTCACGT”. U251 cells were nucleofected with 200nM mirRNAs or 2μg plasmid using the SE Cell Line for 4D-Nucleofector X Kit (Lonza, Basel, Switzerland) according to the manufacturer’s instruction.

### Real-time PCR

Total RNA was isolated from cells or tissues using a mirVana miRNA Isolation Kit (Applied Biosystems/Ambion, Austin, TX, USA) or TRIzol (Life Technology, Grand Island, NY, USA), per the manufacturer’s protocol, and miRNAs from paraffin embedded tissue were isolated by using an miRNeasy FFPE Kit (Qiagen, Hilden, Germany). The cDNA was synthesized using an miScript II RT Kit (Thermo Fisher Scientific Inc., Waltham, MA, USA), per the manufacturer’s instructions. After reverse transcription of the RNA, cDNA was used as a template in PCR reactions using gene-specific primer pairs (Table 2). Primers detecting miR-9-3p, miR-126, miR-572 and U6 were purchased from Ribobio (Guangzhou, China). Real-time PCR was performed on an Applied Biosystems 7500 cycler (Applied Biosystems, CA, USA) using an miScript SYBR Green PCR Kit (Thermo Fisher Scientific Inc.). U6 and glyceraldehyde-3-phosphate dehydrogenase (GAPDH) were used as endogenous controls for miRNA and mRNA expression, respectively. The fold changes of miR-9-3p, miR-126, miR-572 and Herpud1 expression were determined with the ΔΔCt method, and the relative expression of these genes was calculated by the 2^-ΔCT^ method and normalized to U6 or GAPDH.

**Table 2:** The primers for real-time PCR.

Gene	Sequence (5’ to 3’)
Herpud1 forward primer	ATGTACCTGCATCACGTTGG
Herpud1 reverse primer	TGGTTGGGGTCTTCAGTTTC
GAPDH forward primer	CAGCCTCAAGATCATCAGCA
GAPDH reverse primer	TGTGGTCATGAGTCCTTCCA

### Westernblot

Cells were lysed in RIPA buffer. Samples were separated by 12% polyacrylamide gel, and electrotanferred to nitrocellulose. Membranes were blocked with 5% skim milk in TBST and then incubated with anti-HERPUD1 (1:1000, ab73669, Abcam, Cambridge, MA, USA) or anti-GAPDH (1:10000, 10494-1-AP, Proteintech Group, Chicago, IL, USA) antibody at 4°C overnight. Alexa Fluor 790 goat anti-rabbit IgG (1:10000, ab175781, Abcam) was used as secondary antibody. Fluorescent signals were collected using Odyssey LI-COR infrared imaging system (LI-COR, Lincoln, NE, USA). The quantification of westerblot was using Image Studio Lite Ver 3.1 (LI-COR). The expression of Herpud1 was normalized with GAPDH.

### Plasmid constructs and luciferase reporter assays

**Fig 1:**
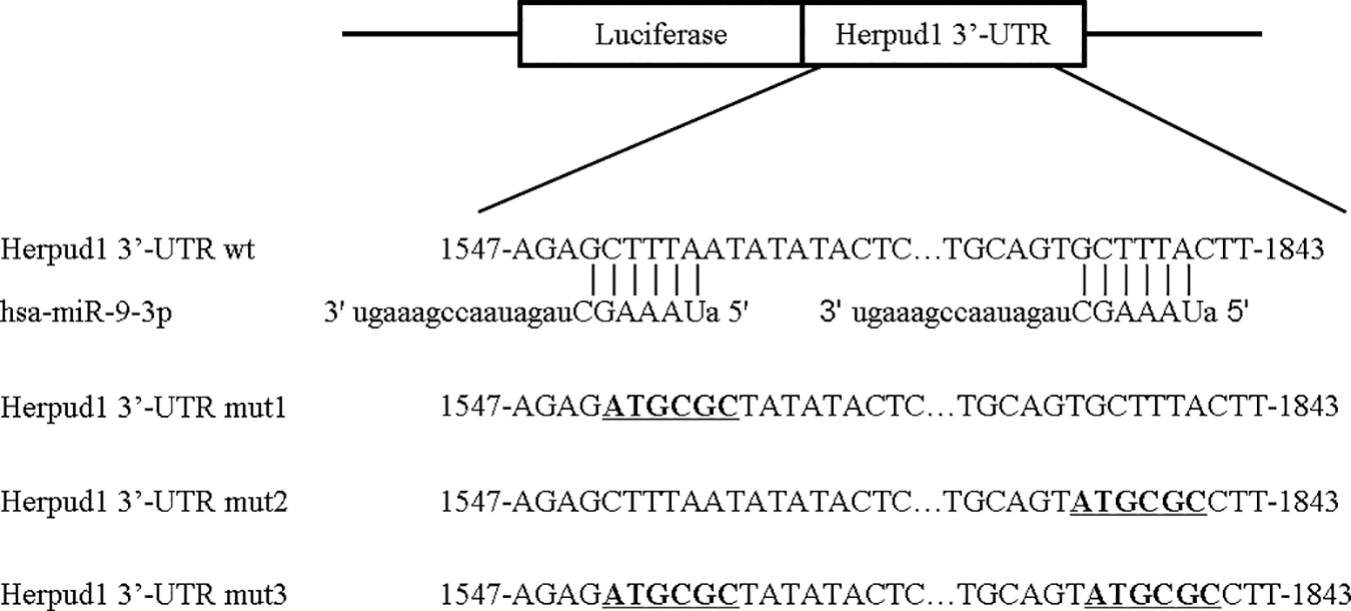
Schematic diagram of the putative miR-9-3p binding site in the UTRs of Herpud1. The seed sequence of miR-9-3p matches the UTRs of Herpud1. The 3 mutated nucleotides of the Herpud1-UTR are in bold and underlined.

The 3’-UTR of Herpud1 and 3 mutation sequences were amplified by PCR with primers containing Mlu I and Hind III restriction sites on each 5’ or 3’ strand. The PCR products were inserted into the Mlu I and Hind III sites of the pMIR-REPORT Luciferase vector and identified through DNA sequencing. The wild-type plasmid containing the 3’-UTR of Herpud1 with the complementary sequence of miR-9-3p (pMIR—Herpud1 3’-UTR wild) and 3 mutant plasmids (pMIR—Herpud1 3’-UTR mut1-3) containing the mutation sequences are in bold and underlined in Fig 1.

For the luciferase reporter assays, 293t cells were seeded on 96-well plates and co-transfected according to the manufacturer’s instructions by using Lipofectamine 2000 (Invitrogen) with 200 ng per well of the luciferase reporter vector, 10 ng per well of pRL-CMV vector (internal control, Promega), and 100 nM per well of miR-9-3p or control (Ribobio). After 48 h, the cells were lysed, and the relative luciferase activity was assessed with a Dual-Luciferase Assay Reporter System (Promega).

### Cell proliferation using Cell Counting Kit-8 (CCK8) assay

Nucleofected U251 cells were seeded in 96-well plates with 3,000 cells per well. The cells were then cultured for 24 h, 48 h or 72 h. The cell proliferation was examined by CCK-8 (Solarbio, Beijing, China) according to the manufacturer’s instructions. Briefly, the supernatant was removed, and 100 μl of DMEM growth medium and 10 μl of CCK8 was added to each well, and the cells were further incubated at 37°C for 2.5 hours. Absorbance was measured at 450 nm using a microplate reader (Rayto, Shenzhen, China).

### Apoptosis assay by flow cytometry

Apoptosis of U251 cells induced by H_2_O_2_ was quantified using a commercially available FITC Annexin V Apoptosis Detection Kit I (556547, BD Biosciences Pharmingen, San Diego, CA, USA)

### Statistical analysis

Data were analyzed with the SPSS 13.0 statistical package (SPSS Inc, Chicago,IL, USA), and real-time PCR data were tested by independent sample t-test. A P value <0.05 was considered to be significant.

## Results

### MicroRNA expression profiling and microarray validation

**Fig 2:**
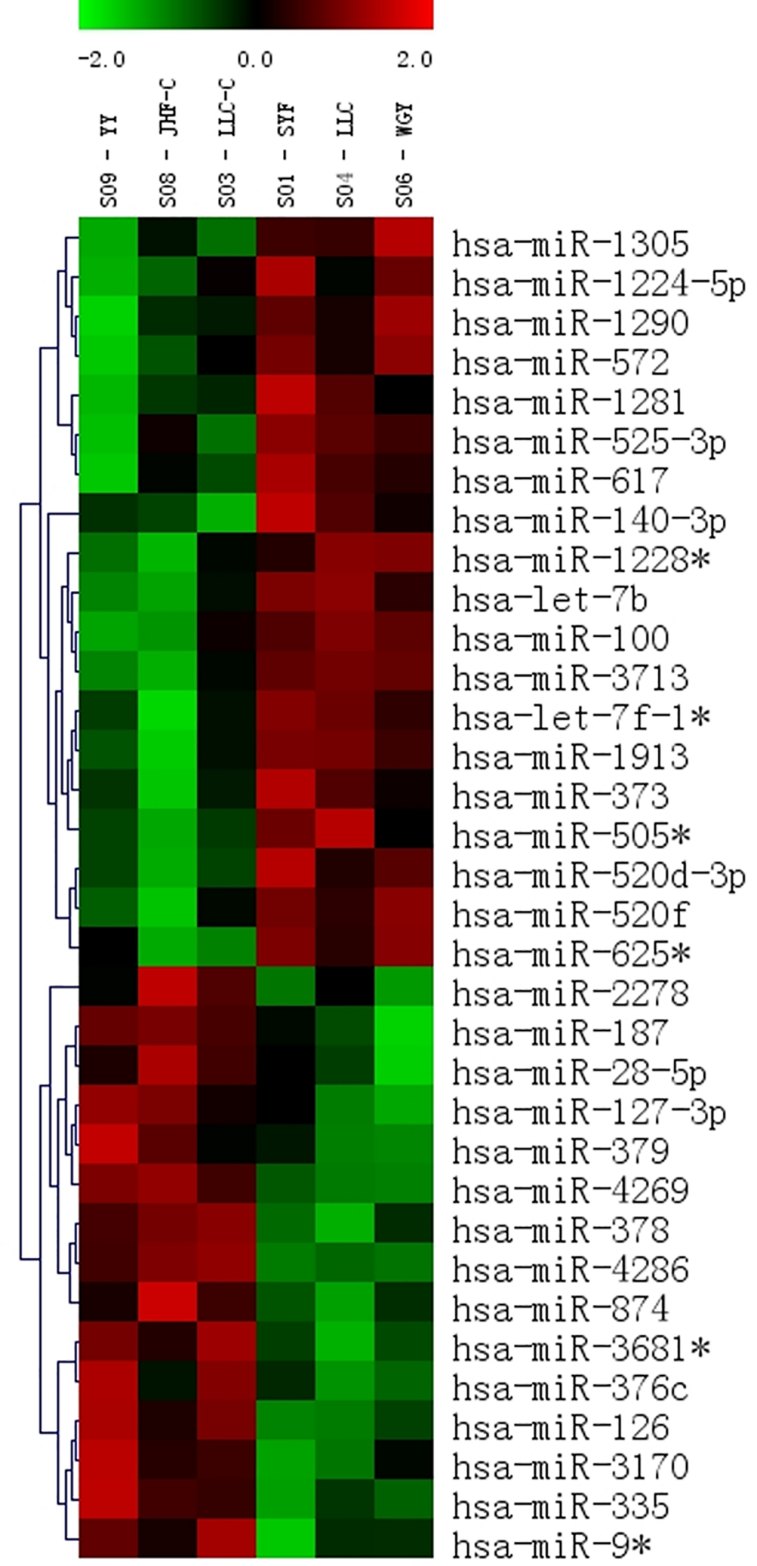
Heat map of differential miRNA expression in glioma and non-tumor tissues. Both down-regulated (red) and up-regulated (blue) miRNAs were identified in glioma tissues (N = 3) versus non-tumor tissues (N = 3).

**Fig 3:**
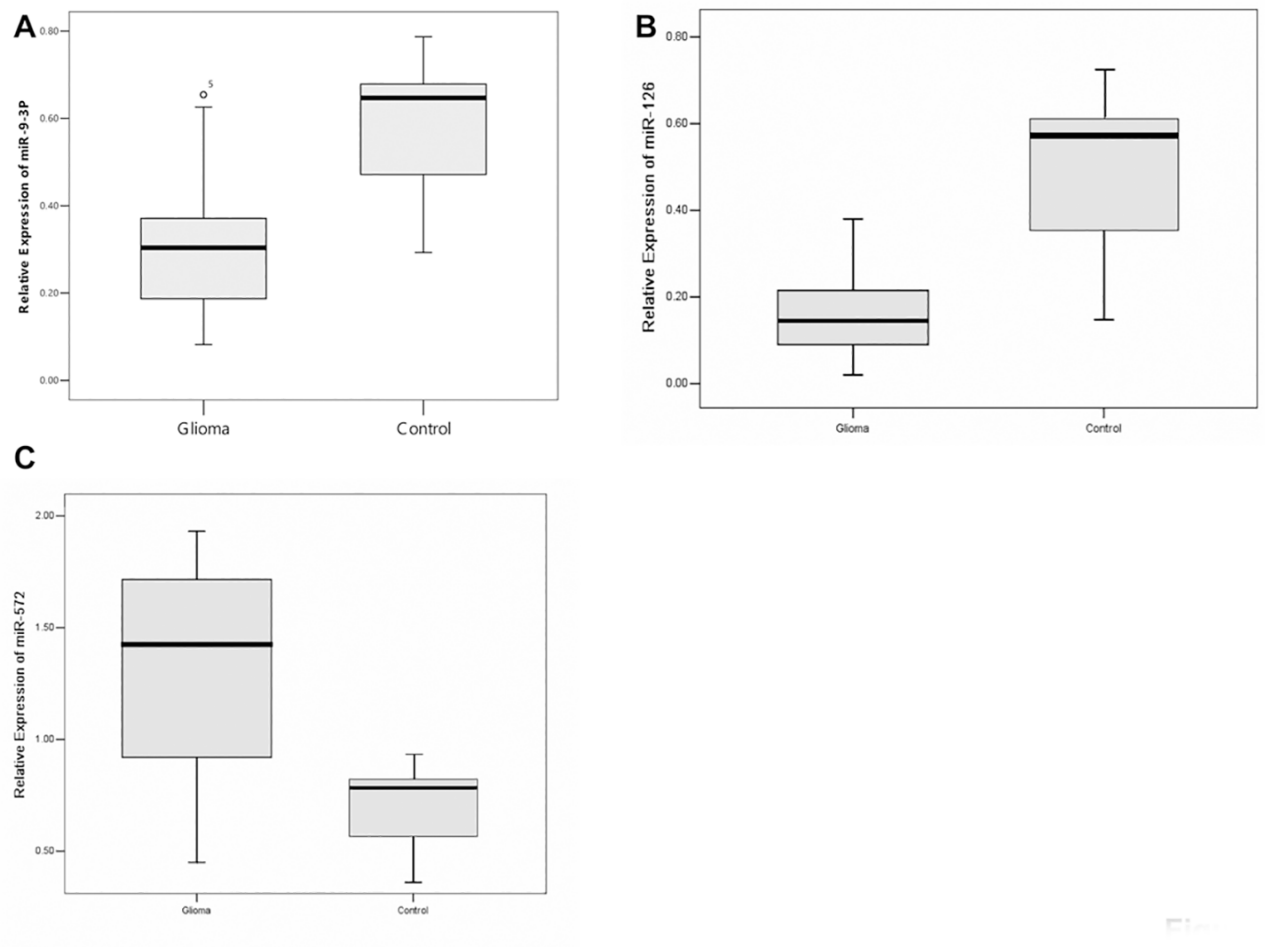
Relative expression levels of miRNAs were measured in 24 glioma samples and 5 control samples by using quantitative RT-PCR with U6 as an internal control (normalized by U6 using 2^-Δct^). The box extends from the 25^th^ to the 75^th^ percentile; the bar in the middle indicates the 50^th^ percentile; the whisker caps indicate the maximum and the minimum values. (A) miR-9-3p expression (P = 0.003); (B) miR-126 expression (P<0.0001); (C) miR-572 expression (P<0.0001).

The microRNA expression profiling patterns in gliomas and non-tumor tissue revealed that 34 of 1145 miRNAs were differentially expressed in human glioma tissue and non-tumor tissue. Among them, 19 miRNAs were over-expressed in glioma tissues, and the remaining 15 were under-expressed, as shown by unsupervised hierarchical clustering without any sample identifiers (Fig 2). We then verified the results of the microarray analysis for miR-9-3p, miR-126, and miR-572 in 24 glioma tissues and 5 controls by using real-time PCR. For miR-9-3p, miR-126, and miR-572 expression, we found a good correlation between the Illumina microarray results (down 1.90-fold, down 2.63-fold, up 2.50-fold, respectively) and the real-time PCR results (average fold change: 1.89, 2.93, 2.05, respectively; Fig 3). U6 was used as an internal control. There was no different expression of miR-9-3p, miR-126, and miR-572 between grade III and IV glioma tissues (data not shown).

### Association of miR-9-3p with clinicopathological characteristics of gliomas

We investigated the association between miR-9-3p expression and the clinicopathological features of glioma. The expression of miR-9-3p was not associated with sex (P = 0.282). However, older patients and patients with higher WHO grade had lower levels of miR-9-3p expression (P = 0.042, P = 0.025, respectively; Table 3). Then we divided glioma tissues into astrocyte, oligodendroglia and mixed cellularity according to which cellular type the tumor cells originated from. The expression of miR-9-3p was not associated with the origin of tumor cells (P = 0.522, Table 3).

**Table 3:** Association between the expression levels of the miR-9-3p and clinicopathological features of glioma tissues.

Clinicopathological features	No.of cases (%) (n = 36)	Expression of miR-9-3p (Mean±Std)	*P* value
Gender			
Male	18 (50.00)	0.29±0.14	*P* = 0.282
Female	18 (50.00)	0.24±0.11	
Age(year)			
<40	23 (63.89)	0.31±0.14	*P* = 0.042
> = 40	13 (36.11)	0.22±0.09	
WHO grade			
I-II	24 (66.67)	0.30±0.13	*P* = 0.025
III-IV	12 (33.33)	0.19±0.08	
Tumor cell origin			
Astrocyte	21(58.33)	0.28±0.17	*P* = 0.522
Oligodendroglia	11(30.56)	0.34±0.21	
Mixed cellularity	4 (11.11)	0.23±0.09	

### MiR-9-3p regulates expression of Herpud1

**Fig 4:**
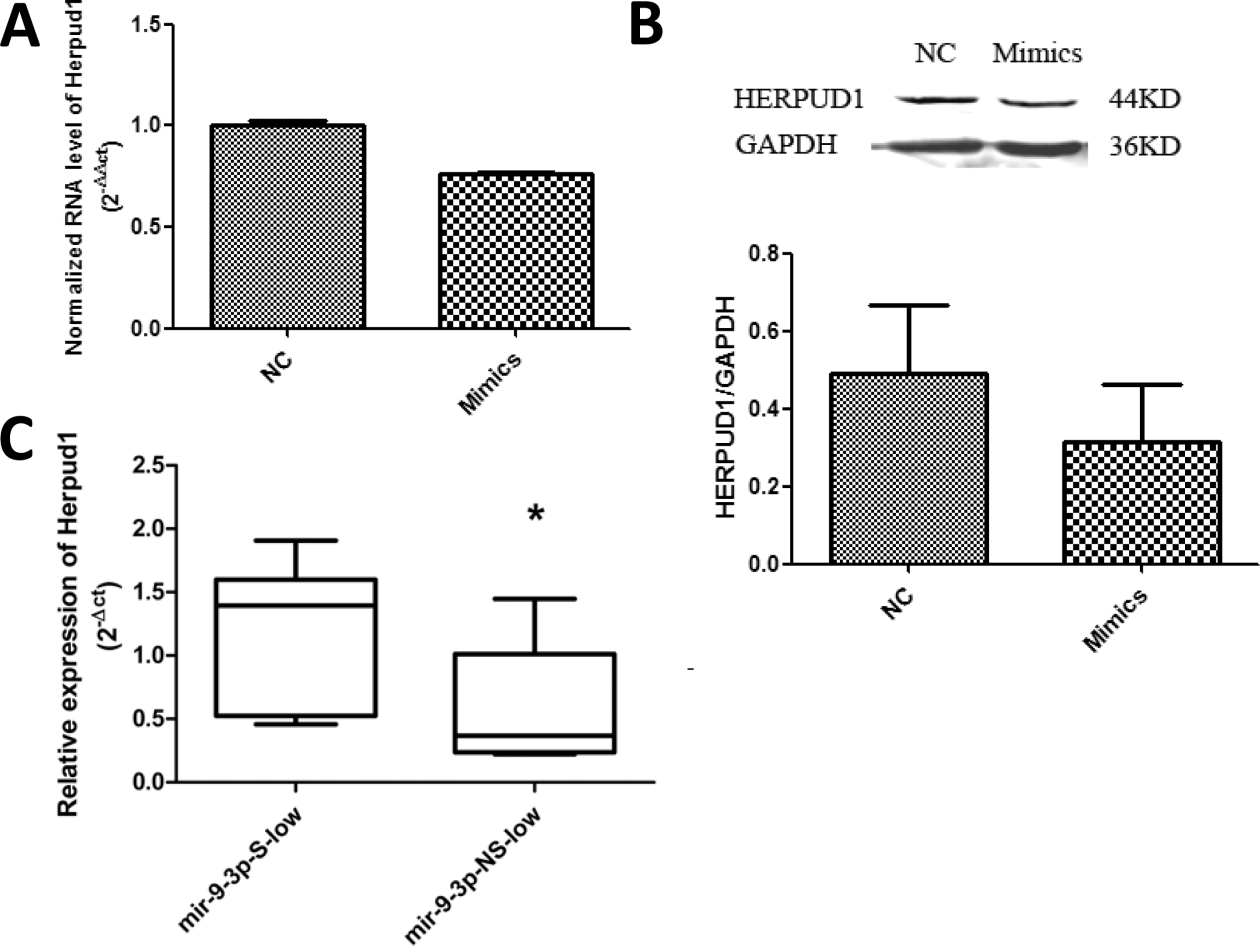
Herpud1 levels in U251 cells and glioma tissues. (A) U251 cells were transfected with 200 nM negative control miRNA (NC) and miR-9-3p mimics. Expression of Herpud1 was normalized by GAPDH using the 2^-ΔΔct^ method. **P<0.01; (B) and the protein level of HERPUD1 was detected by westernblot. (C) Relative expression levels of Herpud1 were measured by using qRT-PCR with GAPDH as an internal control. The box extends from the 25^th^ to the 75^th^ percentile; the bar in the middle indicates the 50^th^ percentile; the whisker caps indicate the maximum and the minimum values. S: significant; NS: not significant. *P<0.05.

We then identified potential miR-9-3p target genes by using Micro-Cosm Target and TargetScan software (miRBase) and found that homocysteine-induced ER stress-inducible, ubiquitin-like domain member 1 (Herpud1) was a potential target gene. In U251 cells, miR-9-3p mimics, compared with a NC (negative control), decreased the mRNA levels (Fig 4A) and the protein level (Fig 4B) of Herpud1. We also examined the expression of Herpud1 in glioma tissues. Herpud1 mRNA expression was higher in tissues with low miR-9-3p expression (P = 0.033; Fig 4C).

### MiR-9-3p binds to the 3’-untranslated region of Herpud1

**Fig 5:**
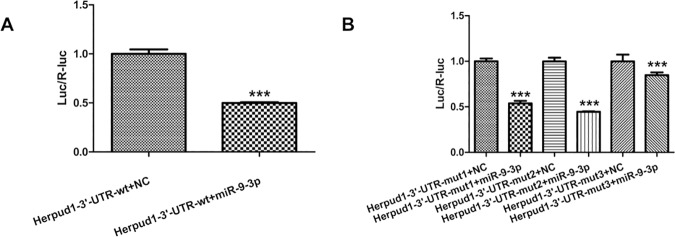
Relative luciferase activities of Herpud1 wild type (wt) UTRs (A) and three mutant UTRs of Herpud1 (B) were obtained by co-transfection of negative control miRNA or miR-9-3p mimics and pRL-CMV plasmid. Relative luciferase activity was calculated as the ratio of firefly/Renilla activities in the cells and normalized to those of the control. The results are presented as mean±SD from three independent experiments. ***P<0.001.

To demonstrate the direct interaction between miR-9-3p and Herpud1 mRNA, we constructed a luciferase reporter system containing 2 binding sites (Herpud1-3’-UTR-wt), one mutated site (Herpud1-3’-UTR-mut1, mut2) or two mutated sites (Herpud1-3’-UTR-mut3). The vectors were co-transfected into 293t cells with miR-9-3p mimics or negative controls. The luciferase activity in the miR-9-3p group was decreased by 50% (P< 0.0001) compared with negative controls (Fig 5A). MiR-9-3p mimics also affected the luciferase activity in cells transfected with the Herpud1-3’-UTR-mut1 and mut2 vectors (P < 0.0001). The inhibition of luciferase activity was greatly decreased in cells transfected with the Herpud1-3’-UTR-mut3 vector (P < 0.0001), although the luciferase activity in the miR-9-3p group also decreased by 15% in cells transfected with the Herpud1-3’-UTR-mut3 vector (Fig 5B). The results indicated that the two predicted sites were the binding sites of miR-9-3p and that there were other binding sites besides the predicted sites that we mutated. These results support the bioinformatics prediction that the 3’-UTR of Herpud1 mRNA may be a target of miR-9-3p.

### MiR-9-3p augments H_2_O_2_ induced apoptosis

**Fig 6:**
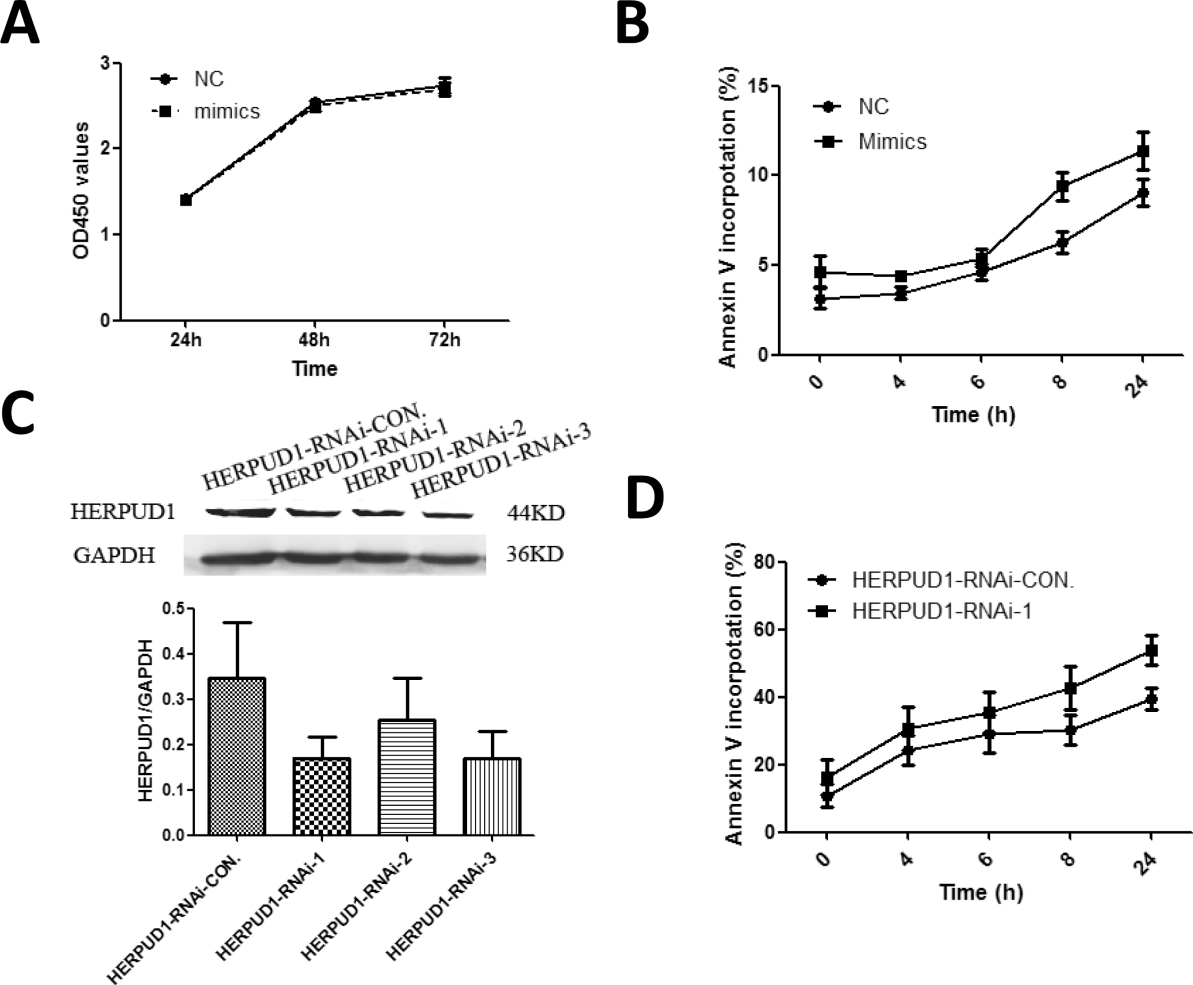
MiR-9-3p augments H_2_O_2_ induced apoptosis through Herpud1. (A) Cell proliferation of transfected U251 cells was determined using CCK8 assay at 24h, 48 and 72h. U251 cells were treated with 500μM H_2_O_2_ for 4, 6, 8 and 24h after transfected with miR-9-3p mimics (B) or HERPUD1-RNAi plasmids (D) for 48h. Apoptotic cells were detected by staining with FITC-Annexin V and propidium iodide. (C) U251 cells were transfected with 2μg HERPUD1-RNAi-CON. and three RNAi plasmids. The protein level of HERPUD1 was detected by westernblot.

First, we investigated whether miR-9-3p mimics affected proliferation of U251 cells. Compared with NC, U251 cells transfected with miR-9-3p mimics did not have effect on proliferation of U251 cells (Fig 6A). U251 cells transfected with miR-9-3p mimics showed more apoptotic cells compared to cells transfected with NC after induced by H_2_O_2_ (Fig 6B). In order to determine whether miR-9-3p regulated apoptosis through Herpud1, three HERPUD1-RNAi plasmids were used to knock down the level of Herpud1 in U251 cells. HERPUD1-RNAi-1 and 3 decreased the protein levels of Herpud1 by about 50% in U251 cells (Fig 6C). HERPUD1-RNAi-1 and 3 was used for further experiment. HERPUD1-RNAi-1 showed better result in the experiment. U251 cells transfected with HERPUD1-RNAi-CON. and HERPUD1-RNAi-1 were treated with H_2_O_2._ The low level of HERPUD1 protein enhanced the apoptosis induced by H_2_O_2_ (Fig 6D). Our results suggested that miR-9-3p augmented apoptosis through down-regulating Herpud1.

## Discussion

In the present study, by using miRNA microarrays, we found decreased expression of miR-9-3p in glioma tissues and identified Herpud1 as the target of miR-9-3p. Several previous studies have reported on the function and targets of miR-9-3p. In hepatocellular carcinoma cells, miR-9-3p reduces lipid accumulation by targeting sirtuin type 1 [27] and inhibits proliferation by targeting PDZ-binding motif (TAZ) [28]. Additionally, miR-9-3p targets β1 integrin and sensitizes breast cancer cells to the MEK1/2 inhibitor AZD6244 [29]. Results from the literature and this study suggest a tumor-suppressing function of miR-9-3p. However, one study has reported that miR-9-3p regulates expression of calmodulin-binding transcription activator 1 (CAMTA1) in CD133+ glioblastoma stem cells [30]. CAMTA1 expression reduces neurosphere formation and tumor growth in nude mice. Our results showed that miR-9-3p augmented apoptosis through down-regulating Herpud1in glioma cells Together, these studies show that miR-9-3p regulates the physiological and pathological process by acting on a variety of targets in different types of cancers. Our results also indicated that the expression of miR-9-3p is associated with patient age and tumor grade.

Herpud1 is a mammalian ubiquitin domain protein, which is strongly induced by the unfolded protein response (UPR) [31,32]. The function of Herpud1 is not fully understood, but accumulating evidence suggests that it has an essential role in ER-membrane-associated protein degradation (ERAD), which translocates ubiquitylated proteins from the endoplasmic reticulum (ER) to proteasomes for degradation [33,34]. Herpud1 has cytoprotective roles against ER stress [35]. Moreover, Herpud1 protects against autophagy by inducing the degradation of Beclin-1 [36]. Recent studies also indentify Herpud1 as cytoprotective factor against oxidative stress in cancer cells [35]. Our microarray results and validation experiments in glioma tissues showed decreased expression of miR-9-3p. In these glioma tissues, expression of Herpud1 was increased compared with controls. In U251 cells with high level of miR-9-3p, mRNA and protein level of Herpud1 was decreased. U251 cells transfected with miR-9-3p mimics showed more apoptotic cells after induced by H_2_O_2_. The similar situation was also observed in HERPUD1-knocking down cells. These results indicated that Herpud1 had protective effect against oxidative stress in glioma cells. The glioma might develop a strategy to protect against oxidative stress through down-regulating miR-9-3p and resulting in up-regulating its target gene Herpud1.

Several studies investigated the interaction of miRNA and ROS in neural disease and gliomas. MiR-34a expression was decreased in human glioma tissues. MiR-34a induced apoptosis in the glioma cell A172 through regulating NOX2 [23]. HIF-2α mRNA and miR-210 expression was increased in hypoxic glioma stem cells (GSCs). Knocking-down of miR-210 inhibited the stemness while induce differentiation and apoptosis in GSCs [37]. One study in MELAS syndrome, a rare neurodegenerative disease caused by mutations in mitochondrial DNA, found that oxidative stress induced expression of miR-9/9*, subsequently decreased the mitochondrial tRNA modification enzymes. MiR-9/9* was implicated in mitochondrial dysfunction[38]. This study combined with ours indicated that miR-9-3p might acting on different targets in different pathological process responded to ROS.

In summary, we found, through microarray analysis and real-time PCR validation, that miR-9-3p has lower expression in gliomas. Expression of miR-9-3p is associated with patient age and tumor grade. Herpud1 is regulated by miR-9-3p and is its target. MiR-9-3p enhanced apoptosis induced by H_2_O_2_ through down-regulating Herpud1.
